# Wealth Inequality and Mental Disability Among the Chinese Population: A Population Based Study

**DOI:** 10.3390/ijerph121013104

**Published:** 2015-10-19

**Authors:** Zhenjie Wang, Wei Du, Lihua Pang, Lei Zhang, Gong Chen, Xiaoying Zheng

**Affiliations:** 1 Institute of Population Research/WHO Collaborating Center on Reproductive Health and Population Science, Peking University, Beijing 100871, China; E-Mails: zhenjie.wang@pku.edu.cn (Z.W.); weidu@pku.edu.cn (W.D.); pang@pku.edu.cn (L.P.); zhang.lei@pku.edu.cn (L.Z.); chengong@pku.edu.cn (G.C.); 2 Laboratory of Neuroscience and Mental Health, Peking University, Beijing 100871, China

**Keywords:** mental disability, wealth inequality, China

## Abstract

In the study described herein, we investigated and explored the association between wealth inequality and the risk of mental disability in the Chinese population. We used nationally represented, population-based data from the second China National Sample Survey on Disability, conducted in 2006. A total of 1,724,398 study subjects between the ages of 15 and 64, including 10,095 subjects with mental disability only, were used for the analysis. Wealth status was estimated by a wealth index that was derived from a principal component analysis of 10 household assets and four other variables related to wealth. Logistic regression analysis was used to estimate the odds ratio (OR) and 95% confidence interval (CI) for mental disability for each category, with the lowest quintile category as the referent. Confounding variables under consideration were age, gender, residence area, marital status, ethnicity, education, current employment status, household size, house type, homeownership and living arrangement. The distribution of various types and severities of mental disability differed significantly by wealth index category in the present population. Wealth index category had a positive association with mild mental disability (*p* for trend <0.01), but had a negative association with extremely severe mental disability (*p* for trend <0.01). Moreover, wealth index category had a significant, inverse association with mental disability when all severities of mental disability were taken into consideration. This study’s results suggest that wealth is a significant factor in the distribution of mental disability and it might have different influences on various types and severities of mental disability.

## 1. introduction

Mental health is an essential state of wellbeing [1]. In transitional China, which has been undergoing rapid socioeconomic development, the prevalence rate of mental disorders had rapidly increased by approximately 17% in a recent study conducted in four provinces in China [2,3]. Many persons with mental disorders also experience disability, which causes long term impairments in personal and social functioning. In 2006, approximately 5.8 million Chinese lived with mental disability, and the national prevalence of mental disability has tripled from 0.2% to 0.6% in 20 years [4].

Among many determinants of disability, socioeconomic status could be measured by income, education, and employment [5,6,7,8]. Previous studies reported that income, education and employment had strong correlations with disability [9]. Poverty, a crucial measurement of socioeconomic status, was also reported having a positive correlation with disability [9,10,11]. However, little evidence has been reported considering the association between scarcity of comprehensive economic possessions and disabilities. In this study, we estimated a diverse range of elements including household assets, income, rurality of residence, and provincial GDP to derive an index of wealth as an indication of wealth status. We aim to investigate the association between wealth index and various types of mental disability, using data from a nationally representative survey [12].

## 2. Methods

### 2.1. Data Source

The second China National Sample Survey on Disability employed a multistage, stratified random cluster sampling scheme, with probability proportional to size, to derive a nationally representative sample. The survey protocol and questions were reviewed by leading national and international experts, and the sampling plan was reviewed by experts from the Division of Statistics of the United Nations [12]. This survey was conducted from 1 April to 31 May 2006. This national survey aimed to describe the prevalence and causes of different types of disabilities, and to explore the socioeconomic and demographic characteristics of people with disabilities in China. The survey covered all provincial administrative areas in mainland China, excluding Hong Kong, Macau and Chinese Taipei. The final sample size represented 1.9 per 1000 non-institutionalized inhabitants of China in 2006. Survey respondents provided consent to participate to the Chinese government. The survey captured visual disability, hearing disability, physical disability, speech disability, intellectual disability and mental disability [12]. The total sample size of the second China National Sample Survey on Disability was 2,526,145, including 23,840 visual disability only; 38,370 hearing disability only; 2510 speech disability only; 48,045 physical disability only; 10,844 intellectual disability only; 11,790 mental disability only; 26,080 multiple disability; and 2,364,666 healthy people. The final analysis examined the population aged 15–64 years including 10,095 with mental disability only and 1,714,303 healthy people.

### 2.2. Ethics

The surveys were approved by the State Council (Guo Ban Fa No. 73 [2004]) and conducted in all province-level administrative regions of mainland China and carried out by the Leading Group of China National Sample Survey on Disability and the National Bureau of Statistics. All survey respondents provided consent to participate in these surveys and clinical diagnosis.

### 2.3. Data Collection Procedures and Data Quality

Pilot studies were conducted in different provinces before the survey period [12]. More than 20,000 interviewers and 6000 doctors of various specialties as well as 50,000 survey assistants participated in this survey. Trained field interviewers used a structured interview questionnaire during data collection to inquire about mental disabilities. Subjects, who responded “yes” to any of the corresponding questions, and all children aged 6 years and under, were assigned to different designated physicians for further disability screening and confirmation. Following the guidelines of diagnostic manuals, designated physicians performed the medical examinations, made a final diagnosis of the disability, if any, then assessed its severity and confirmed the primary cause [12]. Respondents with multiple positive answers were examined by multiple specialists (a separate doctor for each disability). Strict quality control measures were implemented at every step during the survey, from drafting the sample frame to field sampling, from completing the questionnaires to checking the returned forms, and from the data input to checking data quality [12].

### 2.4. Identification of People with Mental Disability

Mental disability was defined and classified by the expert committee of the Second China National Sample Survey on Disability, based on the WHO International Classification of Functioning, Disability and Health (WHO-ICF) [13]. Mental disability was diagnosed by professional psychiatrists according to the International Statistical Classification of Diseases, 10th Revision (ICD-10) and WHO-ICF [13,14]. We defined types of mental disability as organic mental disorders; mental disorders due to psychoactive substance use; schizophrenia, schizotypal and delusional disorders; mood disorders; neurotic stress-related and somatoform disorders; behavior syndromes; disorder of adult personality and behavior; epilepsy; others *i.e.*, undetected types of mental disability. Psychiatrists used the WHODAS II as a scoring tool to assess the severity of the mental disability. Severity of the disability was classified into four categories (mild, moderate, severe and extremely severe) in this study [15]. All the classifications and grading standards, screening methods, diagnosing methods, and relevant scales of disabilities were pretested in pilot studies, and had good reliability and validity.

### 2.5. Study Variable Definition

Mental disability was categorized as binary, *i.e.*, yes or no; survey age and age of mental disability onset, duration of mental disability (*i.e.*, difference between age of mental disability onset and survey age), household size (*i.e.*, number of persons per household), house area (*i.e.*, number of house area in meter square), electricity expenditure (*i.e.*, average number of electricity expenditure per month in kilowatt hours) were set as continuous variables. We categorized marital status as never married, divorced/widowed, or married; ethnicity as Han or others; education level as never attended school, primary school, or junior high school and above; current employment status as employed or unemployed; house type as concrete, masonry/timber and mixed, or bamboo, straw, and other; homeownership as owned or other; residential area as urban or rural; gender as male or female; living arrangement as living with others or living alone.

### 2.6. Wealth Index

We used the principal component analysis to calculate an index of wealth based on an inventory of 10 household assets (*i.e.*, house area (continuous), house type (bamboo, straw, and other: 2; masonry/timber and mixed: 1; concrete: 0), house ownership (other: 1; owned: 0), household income (continuous), television (yes: 1; no: 0), refrigerator (yes: 1; no: 0), telephone (yes: 1; no: 0), washing machine (yes: 1; no: 0), computer (yes: 1; no: 0), electricity expenditure (continuous), current employment status (employed: 1; unemployed: 0), residential area (urban: 1; rural: 0), gender (male: 1; female:0) and China Statistical Year Book derived *per capita* gross regional product (continuous)) [16]. In the principal component analysis, the factors were rotated by orthogonal transformation (varimax rotation) to maintain uncorrelated factors and greater interpretability. Factor-loading matrix for wealth index is presented in Table 1.

**Table 1:** Factor-loading matrix for e wealth index patterns by principal component analysis.

Variables	Factor 1	Factor 2	Factor 3
House area	0.12	0.75	−0.03
House type	−0.57	−0.08	0.10
House ownership	0.14	−0.64	0.06
Household income	0.72	0.06	0.26
Television	0.49	0.29	−0.22
Refrigerator	0.79	−0.004	−0.007
Washing machine	0.68	0.13	−0.09
Telephone	0.56	0.28	−0.16
Computer	0.66	−0.13	0.26
Electricity expenditure	0.74	0.08	0.12
Current employment status	−0.22	0.27	0.64
Residential area	0.66	−0.39	−0.08
Gender	−0.04	0.06	0.67
Per capita gross regional product	0.51	−0.24	0.18
Variance explain (%)	30.2	10.5	8.2

The first three components have the first eigenvalue of 4.23 capturing fully 30.2% of the variance, the second eigenvalue of 1.47 capturing fully 10.5% of the variance, the third eigenvalue of 1.15 capturing fully 8.2% of the variance, respectively. Therefore, the first component is used since it is the one that captures the largest amount of information common to all the items. Weights are determined by factor scores derived from the first principal component. We further categorized the derived wealth index into quintile categories. In this study, the lowest quintile of wealth index presented the poorest status of wealth and the highest quintile of wealth index presented the richest status of wealth.

### 2.7. Statistical Analysis

The trend of the association was assessed with ordinal scores assigned to the quintile categories of the wealth index. Linear regression analysis was used for continuous variables and the Mantel-Haenszel chi-square test was used for categorical variables. The logistic regression analysis was used to estimate the odds ratio (OR) and 95% confidence interval (CI) of wealth index category in association with mental disability. Confounding variables were included as covariates, including survey age, gender, residential area, marital status, ethnicity, education level, current employment status, household size, house type, homeownership and living arrangement. Statistical significance was set at a two-tailed *p* value of <0.05. The statistical analyses were performed using SAS v. 9.2 (SAS Institute, Inc., Cary, NC, USA).

## 3. Results

**Figure 1:**
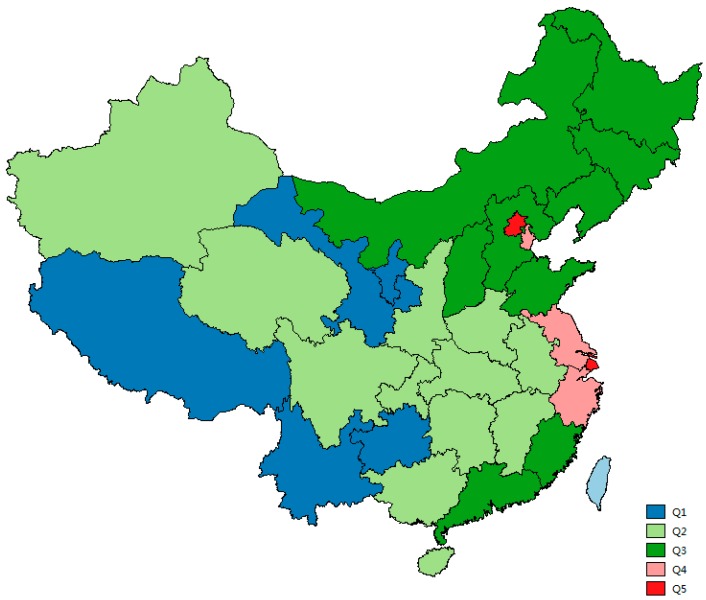
Distribution of wealth index quintiles in China (60% of population live in or above).

**Figure 2:**
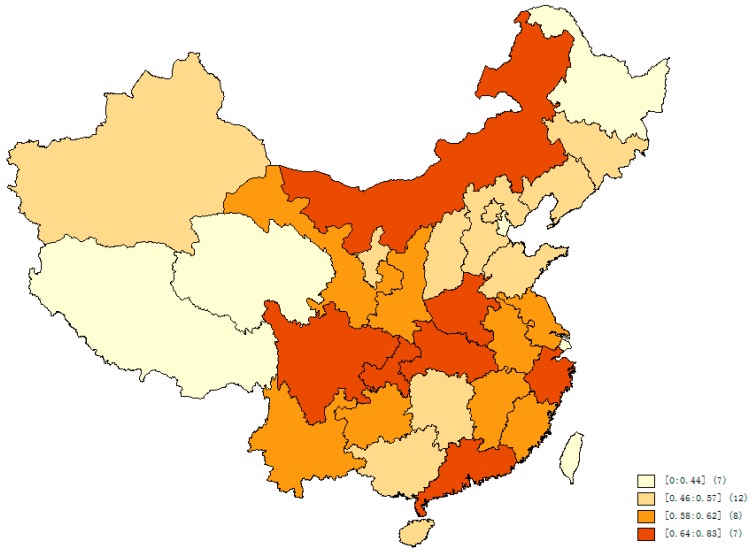
Quartile prevalence of mental disability in China (per 100 persons).

Wealth and mental disability are unequally distributed in China (Figure 1 and Figure 2). Selected characteristics of the study population are summarized in Table 2 and Table 3.

**Figure 3:**
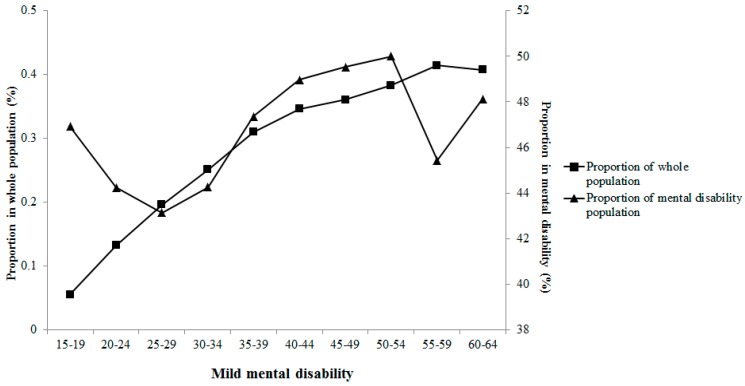
Proportion of mild mental disability in different age groups.

**Figure 4:**
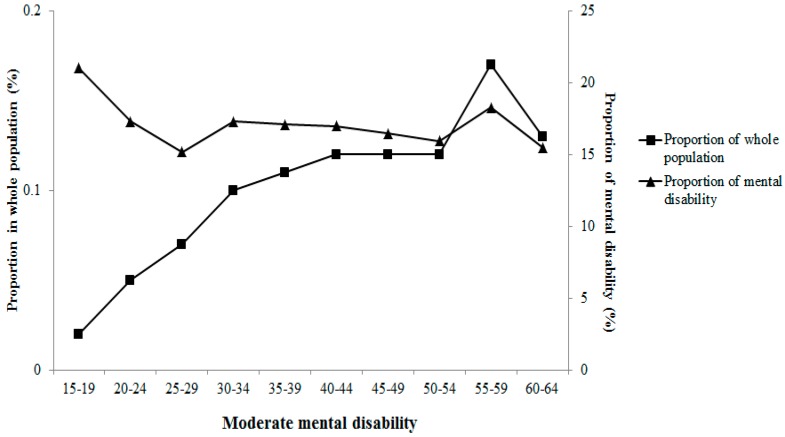
Proportion of moderate mental disability in different age groups.

**Figure 5:**
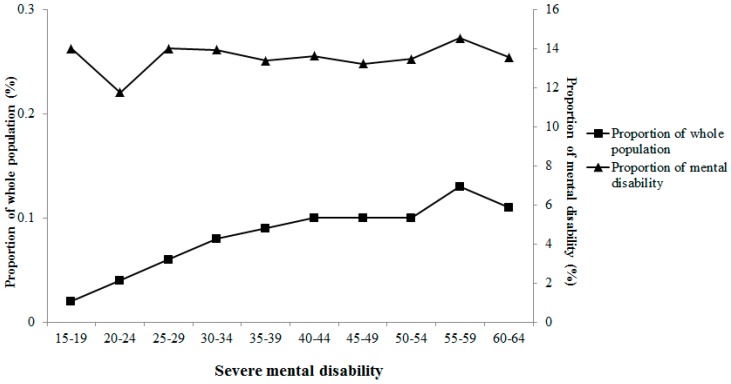
Proportion of severe mental disability in different age groups.

**Figure 6:**
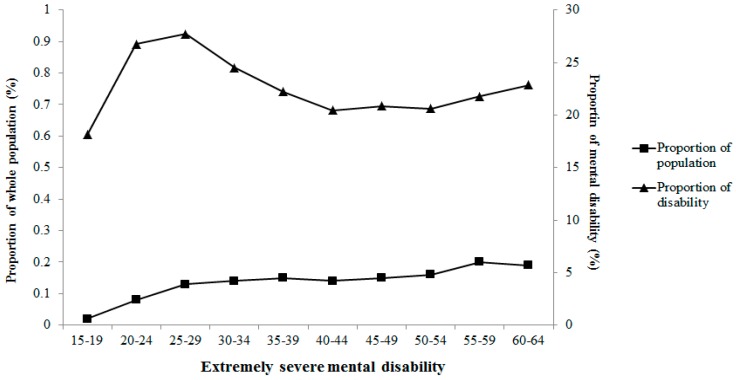
Proportion of extremely severe mental disability in different age groups.

The main mental disability (approximately 60%) was caused by schizophrenia. Moreover, mild mental disability accounted for 47% among persons with mental disability. In the current study, the average survey age of the subjects, onset age of mental disability and education years positively increased with wealth index category, whereas duration of mental disabilities was inversely associated with wealth index category. Severities of mental disability presents varied correlations with survey age in Figure 3, Figure 4, Figure 5 and Figure 6. Severe and extremely severe mental disability presented positive association with survey age, but other severities of mental disability did not show similar associations with survey age.

**Table 2:** Sample size of study population by age groups.

	Mental Disability Severities, *n*					Total Population, *n*
	Mild	Moderate	Severe	Extremely Severe	Total, *n*	
15–19	114	51	34	44	243	206,371
20–24	192	75	51	116	434	145,163
25–29	302	106	98	194	700	154,211
30–34	502	196	158	278	1134	200,582
35–39	716	258	202	336	1512	231,740
40–44	759	263	211	317	1550	219,952
45–49	585	194	156	246	1181	162,815
50–54	691	220	186	285	1382	181,013
55–59	538	216	172	258	1184	130,528
60–64	373	120	105	177	775	92,023
Total	4772	1699	1373	2251	10,095	1,724,398

**Table 3:** Socioeconomic characteristics of the Chinese population by wealth index quintiles ^a^.

Variable	Wealth Index					*p _trend_*
	Q1 (Lowest)	Q2	Q3	Q4	Q5 (Highest)	
Sample size of total population, *n*	344,880	344,880	344,879	344,880	344,879	
Sample size of mental disability, *n*	4017	2208	1618	1274	978	
Age of mental disability onset, mean (years)	27.9	28.4	29.1	30.0	32.0	<0.01
Duration of mental disability, mean (years)	14.9	13.8	13.6	13.4	12.7	<0.01
Male, %	53.8	50.7	49.3	48.9	47.4	<0.01
Marital status, %						
Never married	22.3	20.6	20.5	17.7	19.3	<0.01
Divorced/widowed	5.2	3.1	2.7	3.1	2.8	<0.01
Married	72.5	76.3	76.8	79.2	77.9	<0.01
Urban, %	2.6	9.0	23.0	53.8	88.4	<0.01
Han ethnicity, %	75.4	88.6	92.4	93.4	94.5	<0.01
Education, %						
Never attended school	19.8	10.8	7.4	4.5	1.6	<0.01
Primary school	41.8	34.6	27.5	19.1	8.3	<0.01
High school and above	38.4	54.7	65.1	76.5	90.1	<0.01
House type						
Bamboo, straw, and other	44.2	17.4	6.5	2.1	0.3	<0.01
Masonry/timber and mixed	54.9	79.4	85.7	85.9	74.7	<0.01
concrete	0.8	3.2	7.9	12.0	25.1	<0.01
Homeownership owned, %	97.3	94.1	89.7	85.5	82.1	<0.01
Living with others, %	96.8	98.3	98.5	98.3	98.5	<0.01
Employed, %	88.1	84.2	78.5	70.2	61.1	<0.01
Last year income, mean (Yuan)	6092	9488	12753	17256	34373	<0.01

^a^ Based on linear regression analysis for continuous variables and the Mantel-Haenszel chi-square test for categorical variables, with ordinal scores assigned to the quintile categories.

The characteristics of the mental disability population in this study are summarized by different types and severities across wealth index category in Table 4 and Table 5. Various types of mental disability presented different associations with wealth index. For example, schizophrenia, schizotypal and delusional disorders, and epilepsy were negatively associated with wealth index, but other types of mental disability were positively associated with the wealth index category. Although disorders of adult personality and behavior also exhibited a positive association with wealth index category, this association was not statistically significant. Moreover, mild mental disability was positively associated with the wealth index category, whereas extremely severe mental disability exhibited an inverse association.

**Table 4:** Characteristics of different types of mental disability by wealth index quintiles ^a^.

Variable	Wealth Index					*p _trend_*
	Q1 (Lowest)	Q2	Q3	Q4	Q5 (Highest)	
Types of mental disability						
Organic mental disorders, *n*	227	121	98	90	48	
Age of mental disability onset, mean (years)	25.8	28.8	31.1	34.8	35.0	<0.01
Duration of mental disability, mean (years)	16.4	14.7	13.6	12.7	12.9	0.01
Mental disorders due to psychoactive substance use, *n*	96	63	54	46	33	
Age of mental disability onset, mean (years)	35.8	37.8	33.9	35.4	40.2	0.42
Duration of mental disability, mean (years)	12.7	12.1	12.9	12.9	9.5	0.36
Schizophrenia, schizotypal and delusional disorders, *n*	2731	1460	1016	796	660	
Age of mental disability onset, mean (years)	28.2	28.1	28.3	29.0	30.6	<0.01
Duration of mental disability, mean (years)	14.5	13.5	14.0	13.9	13.4	0.01
Mood disorders, *n*	187	153	124	101	107	
Age of mental disability onset, mean (years)	32.1	34.1	35.3	37.3	38.4	<0.01
Duration of mental disability, mean (years)	12.3	11.4	9.6	8.5	8.7	<0.01
Neurotic stress-related and somatoform disorders, *n*	194	128	107	91	65	
Age of mental disability onset, mean (years)	34.5	36.3	36.9	36.9	38.4	0.01
Duration of mental disability, mean (years)	11.9	11.3	9.6	9.9	9.3	0.01
Behavior syndromes, *n*	20	12	14	9	8	
Age of mental disability onset, mean (years)	29.4	29.6	41.4	43.9	33.3	0.04
Duration of mental disability, mean (years)	13.5	16.5	6.3	8.7	10.1	0.08
Disorder of adult personality and behavior, *n*	48	15	5	12	11	
Age of mental disability onset, mean (years)	24.9	21.5	19.4	19.3	23.1	0.34
Duration of mental disability, mean (years)	17.9	19.8	16.2	12.9	13.1	0.12
Epilepsy, *n*	371	194	150	101	41	
Age of mental disability onset, mean (years)	20.0	19.3	20.5	19.5	19.3	0.88
Duration of mental disability, mean (years)	19.6	17.8	17.9	18.0	19.7	0.27
Others, *n*	143	62	50	28	5	
Age of mental disability onset, mean (years)	28.6	23.9	28.2	22.2	36.2	0.33
Duration of mental disability, mean (years)	15.5	19.0	14.5	15.2	5.6	0.39

^a^ Based on linear regression analysis for continuous variables and the Mantel-Haenszel chi-square test and Mantel-Haenszel exact chi-square test for categorical variables, with ordinal scores assigned to the quintile categories.

**Table 5:** Characteristics of different severities of mental disability by wealth index quintiles ^a^.

Variable	Wealth Index					*p _trend_*
	Q1 (Lowest)	Q2	Q3	Q4	Q5 (Highest)	
Mild, *n*	1782	1033	816	630	511	
Age of mental disability onset, mean (years)	29.4	30.0	30.5	31.7	33.4	<0.01
Duration of mental disability, mean (years)	12.8	12.8	12.3	11.8	11.4	<0.01
Moderate, *n*	652	383	272	199	193	
Age of mental disability onset, mean (years)	27.7	28.4	27.7	29.2	31.1	0.002
Duration of mental disability, mean (years)	14.8	14.1	13.9	13.8	14.6	0.37
Severe, *n*	582	259	215	186	131	
Age of mental disability onset, mean (years)	27.4	28.1	29.8	28.3	30.1	0.01
Duration of mental disability, mean (years)	15.7	14.2	14.1	14.9	13.1	0.02
Extremely severe, *n*	1001	533	315	259	143	
Age of mental disability onset, mean (years)	25.8	25.5	26.1	27.7	30.0	<0.01
Duration of mental disability, mean (years)	16.4	16.1	16.3	15.9	14.2	0.08

^a^ Based on linear regression analysis for continuous variables and the Mantel-Haenszel chi-square test for categorical variables, with ordinal scores assigned to the quintile categories.

**Table 6:** Associations of wealth index and mental disability among the Chinese population.

Regression Models	Wealth Index	OR (95% CI)
Model 1 ^a^	Q1 (lowest)	1.00 (reference)
	Q2	0.53 (0.51–0.56)
	Q3	0.36 (0.34–0.39)
	Q4	0.23 (0.22–0.25)
	Q5 (highest)	0.15 (0.13–0.16)
***p _trend_***	**<0.01**	
Model 2 ^b^	Q1 (lowest)	1.00 (reference)
	Q2	0.53 (0.50–0.56)
	Q3	0.34 (0.31–0.36)
	Q4	0.21 (0.19–0.22)
	Q5 (highest)	0.13 (0.12–0.14)
***p _trend_***	**<0.01**	

Abbreviations: CI = confidence interval; OR = odds ratio. ^a^ Adjusted for age, gender, residence area. ^b^ Adjusted for age, gender, residence area, marital status, ethnicity, education, current employment status, household size, house type, homeownership, and living arrangement.

The association between mental disability and wealth index category is presented in Table 6. Wealth index was negatively associated with the odds ratio of mental disability. Although it showed a significant trend of an inverse association with mental disability, it more obviously decreased the OR of mental disability in the highest quintile of wealth index compared with those at the lowest quintile.

## 4. Discussion

In the present study, the average household income was approximately $2-per-day in the lowest wealth index quintile of the whole study population. Although it was slightly higher than $1.25-per-day line or $1.50-per-day line [17,18], it could represent the poorest people in China. As several epidemiological studies have suggested, there was an increasing risk of mental disability associated with poverty [10,11]. We observed that disadvantaged wealth status was associated with an increased odds ratio of living with mental disability among the Chinese population. Our results also indicated that there was a decreasing trend in mental disability, which suggested that high wealth status was a protective factor for mental disability. Therefore, wealth appeared in this study as a socioeconomic determinant of mental disability among the Chinese population.

Moreover, living in low socioeconomic position could elevate the risk of disability, whereas having a disability could elevate the risk of living in low wealth status [10]. Our study found that extremely severe mental disability was inversely associated with wealth. This result may be due, in part, to the mutually causal nature of low wealth status and mental disability, which is consistent with previous studies [10,11]. Because people who were economically disadvantaged suffered from malnutrition and a lack of adequate access to health services, including maternal care and trauma services [19], they were more likely to suffer from disabilities, which further enhanced their exclusion and marginalization by reducing their opportunities to contribute productively to their households and communities, which in turn increased their risk of the lowest income [19]. Interestingly, we also observed that mild mental disability was positively associated with wealth. This interesting and unique result may be due, in part, to the fact that China is one of the most populous and dynamic societies in the world, the accompanying pressure contributing to more mental health problems in healthy people.

Additionally, schizophrenia accounted for the majority of mental disability cases in the current study. The lowest income and lower social class have long been linked to higher rates of schizophrenia [20]. We observed consistent associations between wealth and schizophrenia-caused disability. However, we also observed that wealth was positively associated with mental disability caused by various conditions such as organic mental disorders, mental disorders due to psychoactive substance use, *etc*, other than schizophrenia and epilepsy. These findings imply that wealth might have differentiated effects on mental disability caused by different conditions.

Considering wealth is a complex economical factor, there are multiple measures of wealth based on income or socioeconomic factors. In the study reported here, we tried to combine all collected economic information to evaluate wealth by using principal components analysis, because principal components analysis provided plausible and defensible weights for an index of assets to serve as a proxy for wealth as a previous study suggested [16]. Furthermore, the current wealth index was developed by using household asset variables, income, expenditure data, and other possible or determining variables related with wealth income disparities in China [21,22], unlike previous studies which only used household asset variables without income or expenditure data to estimate wealth [16]. Therefore, the current wealth index could sufficiently and accurately present the economic status in China. Moreover, the current lowest wealth index category was slightly different from poverty, but it was much more useful and valuable than poverty because it included comprehensive economic information, unlike poverty, which only depends on income.

A limitation of this study is its cross-sectional study design, which cannot provide indisputable information on the relationships between wealth and mental disability. Additionally, the wealth index’s variables were selected based on unique Chinese characteristics and previous researches that should be noted for further studies. Major strengths of the current study are the large sample size and the representativeness of the sample, which covered all the provincial administrative areas in mainland China. In addition, every subject in the selected households was interviewed face to face at data collection. Although the present study has some weaknesses, standardized quality control schemes were in place during the field implementation, such as training of the interviewers and crosschecking returned surveys by contacting survey participants, which resulted in little response bias.

## 5. Conclusions

Wealth was related to mental disability among the Chinese population, especially for those people who lived in the low economic status category. Furthermore, wealth might also have various influences on the distributions of mental disability types and severities by affecting nutritional status, or access to health services. Considering China is undergoing social and economic transition, these unique and interesting results will enhance the understanding of mental disability in the context of wealth, and help government adjust strategies on improving the life of people with different types and severities of mental disability.
